# Mechanism of paraventricular nucleus H_2_S on PERK/TXNIP/NLRP3 pathway in male spontaneously hypertensive rats

**DOI:** 10.14814/phy2.70861

**Published:** 2026-04-10

**Authors:** Yan‐Feng Liang, Shu‐Yue Chen, Hui Wang, Han‐Jun Song, Bao‐Cheng Zhang, Ming‐Fu Wang, Chao Lv, Xiao‐Yu Wang, Shuai Zhang, Huai‐Yu Xing, Quan Yuan, Hong‐Bin Li, Xin‐Rui Ding, Zhang‐Ran Xing, Xin‐Yi Han, Dong‐Dong Zhang

**Affiliations:** ^1^ School of Basic Medicine Jiamusi University Jiamusi China; ^2^ Key Laboratory of Microecology‐Immune Regulatory Network and Related Diseases School of Basic Medicine Jiamusi University Jiamusi China; ^3^ Provincial Demonstration Center for Experimental Teaching of Basic Medicine Jiamusi University Jiamusi China; ^4^ School of Rehabilitation Medicine Jiamusi University Jiamusi China

**Keywords:** hydrogen sulfide, hypertension, paraventricular nucleus, PERK/TXNIP/NLRP3, pro‐inflammatory cytokines

## Abstract

Hydrogen sulfide (H_2_S) exhibits anti‐inflammatory, anti‐apoptotic, and antioxidant effects. However, the central nervous system regulatory mechanisms of H_2_S in hypertension remain unclear. This study aimed to investigate the regulatory effects of H_2_S on the PERK/TXNIP/NLRP3 pathway and the paraventricular nucleus (PVN) inflammatory responses by injecting S‐adenosylmethionine (SAMe, an endogenous H_2_S agonist) or the PERK inhibitor GSK2606414 (GSK) into the PVN of spontaneously hypertensive rats (SHR). Additionally, we employed a lipopolysaccharide (LPS)‐induced cell inflammation model and intervened with SAMe or the NLRP3 activator BMS‐986299 (BMS) to further validate the relevant molecular mechanisms. Relevant indicators were analyzed using tail artery blood pressure measurement, CCK‐8 assay, ELISA, immunofluorescence, western blot, and real‐time quantitative polymerase chain reaction (RT‐qPCR). This study found that H_2_S in the PVN improves spontaneous hypertension by inhibiting the PERK/TXNIP/NLRP3 pathway and reducing sympathetic activity. Cell experiments further confirmed that the inhibitory effect of H_2_S on this pathway is the key mechanism underlying its anti‐inflammatory protective effects.

## Graphical abstract

H^2^S in PVN alleviates hypertension via PERK/TXNIP/NLRP3 inhibition and sympatholysis.

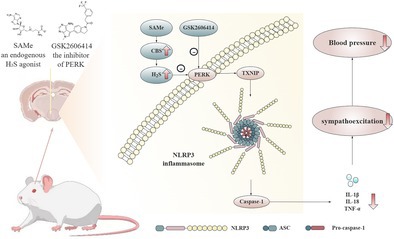

## 1 INTRODUCTION

In clinical practice, hypertension is the most frequent kind of chronic disease. It is the major risk factor for the development of coronary heart disease and stroke in our population. Primary hypertension is the most common type of hypertensive disease, and its incidence has been steadily increasing in recent years (Haseler & Sinha, 2022). Although the molecular mechanism of primary hypertension in the central nervous system (CNS) is not fully elucidated, there is growing evidence that it has a direct relationship with the inflammatory response (Shi & Yong, 2025; Wei et al., 2024).

Hydrogen sulfide (H_2_S), an endogenous gasotransmitter, has been proven by a large number of studies to have anti‐inflammatory, anti‐apoptotic, and antioxidant effects. It has enormous potential in the treatment of many cardiovascular ailments (Cui et al., 2025; Doiron et al., 2025; Kumar, 2023). A crucial central nucleus responsible for the autonomic and endocrine responses is the PVN of the hypothalamus. It acts as a major hub for the regulation of sympathetic nerve function, and it has a very important role in the development of hypertension (Faber et al., 2023; Sotozawa et al., 2024). A large number of studies have shown that various neurohormonal substances in the PVN, such as pro‐inflammatory cytokines (PICs), reactive oxygen species (ROS), and components of the renin‐angiotensin system (RAS), are involved in the pathophysiological process of hypertension (Chen, Lin, et al., 2022; Zheng et al., 2025). The results of previous studies conducted by our group showed that H_2_S in PVN could reduce hypertension by inhibiting inflammatory responses and oxidative stress (Liang et al., 2024). However, the mechanism by which H_2_S in the PVN modulates the inflammatory response to lower blood pressure is unknown.

Protein kinase R‐like Endoplasmic Reticulum kinase (PERK) is one of the sensor molecules involved in Endoplasmic Reticulum Stress (ERS). When endoplasmic reticulum stress occurs, the cells start the Unfolded Protein Response (UPR) to restore the homeostasis of the endoplasmic reticulum. However, if ERS persists for a prolonged period or is too intense, it can contribute to inflammatory responses (Ren et al., 2021). After ESR activates the unfolded protein response, it promotes the transcription of thioredoxin‐interacting protein (TXNIP) via PERK, inhibits the degradation of TXNIP mRNA, and activates the NOD‐like receptor protein 3 (NLRP3) inflammatory complex. Pro‐caspase‐1 is cleaved into its active subunits, p10 and p20. This activated caspase‐1 subsequently processes and promotes the release of the pro‐inflammatory cytokines IL‐1β and IL‐18, a process that initiates a cascade of inflammatory responses (Molla et al., 2020). Research has demonstrated that in the mouse model of hypertension caused by high salt intake, there was a significant increase in the expression of the inflammatory body components NLRP3, ASC, and pro‐caspase‐1, along with a marked elevation in the secretion of IL‐1β. Knockout of the ASC gene in mice could terminate the above changes and improve pulmonary hypertension (Li et al., 2021). Other studies have shown that H_2_S modulates PERK expression (Song et al., 2024). Exogenous administration of sodium hydrosulfide (NaHS) has been demonstrated to modulate TXNIP and the NLRP3/Caspase‐1/IL‐1β inflammatory pathway protein expression (Bao et al., 2023). Therefore, we hypothesize that the anti‐inflammatory and blood pressure‐lowering mechanism of H_2_S may be related to the PERK/TXNIP/NLRP3 pathway.

The effects of H_2_S in the paraventricular nucleus on the PERK/TXNIP/NLRP3 pathway in spontaneously hypertensive rats were studied in this experiment. Our aim was to study the molecular mechanism of H_2_S in hypertension to further elucidate the central mechanism of hypertension and identify potential targets for the prevention and treatment of hypertension.

## 2 MATERIALS AND METHODS

### 2.1 Experimental animals and protocol

The present study included healthy male Wistar‐Kyoto (WKY) rats and spontaneously hypertensive rats (SHRs) aged 8 weeks and weighing 260–280 g, purchased from Beijing Vital River Laboratory Animal Technology Company. The rats were housed under specific pathogen‐free conditions with constant temperature and humidity, maintained on a 12‐h light‐dark cycle and had free access to water and a standard commercial rodent diet (Shenyang Qianmin Feed Co., Ltd., QM‐RM‐M‐10; China). All procedures were conducted following the Guide for the Care and Use of Laboratory Animals (NIH Publication, 8th edition, 2011), and all experimental procedures were approved by the Biological and Medical Ethics Committee of the School of Basic Medical Sciences, Jiamusi University. WKY and SHR were randomly divided into 5 groups (*n* = 14): (1) WKY + vehicle group; (2) SHR + vehicle group; (3) SHR + SAMe (S‐adenosylmethionine, an endogenous H_2_S agonist) group; (4) SHR + GSK (GSK2606414, the PERK inhibitor) group; (5) SHR + SAMe+GSK group. Rats in each group were anesthetized with 3% pentobarbital sodium (50 mg/kg) via intraperitoneal injection, and their heads were fixed in a stereotaxic device. According to the brain stereotaxic atlas, bilateral PVN cannulation was performed at a position approximately 1.8 mm posterior to the fontanel, 0.4 mm lateral to the centerline, and 7.9 mm ventral to the ventral side level. The osmotic pump (ALZET, model 2006) was implanted subcutaneously in the posterior neck and connected to the other end of the catheter. SAMe (2 nmol/h, Sigma‐Aldrich, SML3851; USA) was continuously microinjected into the PVN of rats in the SHR + SAMe group and the SHR + SAMe+GSK group. Concurrently, GSK (10 nmol/h, MedChemExpress, HY‐18072; USA) was injected into the SHR + GSK group and the SHR + SAMe+GSK group, respectively. The WKY + vehicle group and the SHR + vehicle group were injected with an equivalent dose of artificial cerebrospinal fluid.

### 2.2 Non‐invasive blood pressure measurement

Blood pressure measurements were performed using a BP‐100 tail artery blood pressure meter in a quiet environment, and the mean arterial pressure (MAP) was recorded for each rat. One week before the formal measurements, rats were trained daily to adapt to the tail‐cuff procedure and the experimental environment. During measurements, awake rats were gently restrained in a rat‐specific holder. After the animals calmed down, they were placed on a heating plate pre‐set at 36°C to stabilize the pulse signal. The temperature was then adjusted to 34°C, and the tail was gradually pressurized while the animal remained calm. Each rat was measured eight times consecutively, and the average value was taken as the final MAP for that rat. A total of five measurements were performed: one baseline measurement before drug administration, followed by weekly measurements during the four‐week treatment period. All measurements were conducted at the same time of day (between 9:00 am and 11:00 am) to minimize circadian variations.

### 2.3 Tissue collection

After 4 consecutive weeks of drug administration, rats were anesthetized via intraperitoneal injection of 3% pentobarbital sodium (100 mg/kg). Following anesthesia, blood samples were collected from the abdominal aorta. Subsequently, 4 rats were randomly selected from each group, and the chest was quickly opened to expose the heart. A perfusion needle was inserted into the left ventricle, and rats were transcardially perfused sequentially with 500 mL of physiological saline and 300 mL of 4% paraformaldehyde. After the liver and limbs became stiff, the whole brain was carefully isolated and fixed in 4% paraformaldehyde until further use. The remaining rats were subjected to rapid brain tissue dissection for subsequent analyses.

### 2.4 Cell culture and treatment

PC12 cells (Pheochromocytoma of the Rat Adrenal Medulla cells, Shanghai Zhongqiao Xinnzhou Biotechnology Co., Ltd.) were cultured in high‐glucose Dulbecco's Modified Eagle Medium (DMEM, Hyclone, SH30022.01; USA) supplemented with 10% fetal bovine serum (FBS, Shanghai VivaCell Biotech Co., Ltd., C04001; China) and 1% penicillin–streptomycin solution (Shanghai Beyotime Biotechnology Co., Ltd., C0222; China). Cells were used at passages 5–10 and maintained in 25 cm^2^ plastic culture flasks at 37°C in a humidified atmosphere of 5% CO_2_. For experiments, cells were seeded into 6‐well plates at a density of 5 × 10^5^ cells per well and cultured for 24 h before treatment. The experimental groups were as follows: (1) control group; (2) LPS group; (3) LPS + BMS (BMS‐986299, the NLRP3 activator) group; (4) LPS + SAMe group; (5) LPS + BMS + SAMe group. The LPS group, the LPS + BMS group, the LPS + SAMe group, and the LPS + BMS + SAMe group were treated with LPS (1 μg/mL, Sigma‐Aldrich, L2880; USA) for 24 h. Subsequently, the LPS + BMS group and the LPS + BMS + SAMe group were treated with BMS (1 μM, MedChemExpress, HY‐18072; USA) for 1 h. The LPS + SAMe group and the LPS + BMS + SAMe group were additionally treated with SAMe (10 μM) for 1 h.

### 2.5 CCK‐8 assay

Cell viability was detected using the CCK‐8 assay. The experimental procedure was as follows: Cells were digested, centrifuged, and resuspended in complete medium, then seeded at a density of 5000 cells per well in a 96‐well plate. After 24 h of incubation, the cells were treated with varying concentrations of BMS and SAMe (0, 0.1, 1, 5, 10, and 100 μM). A 10 μL CCK‐8 solution (Shanghai Beyotime Biotechnology Co., Ltd., C0038; China) was added per well, and the absorbance at 450 nm was measured using an enzyme‐labeled instrument. Each concentration was tested in triplicate, and the mean value was used for analysis. Three independent biological replicates were performed.

### 2.6 ELISA analysis

PVN tissue samples were immediately and rapidly dissected on ice. Tissue samples were weighed and homogenized in PBS (pH 7.4) supplemented with protease inhibitors. The homogenates were then centrifuged at 3000×*g* for 10 min at 4°C, and the supernatants were carefully collected for H_2_S assay. Plasma NE levels were measured using a commercially available rat ELISA kit (Invitrogen, EEL010; USA). H_2_S levels in the PVN were measured using a rat hydrogen sulfide ELISA kit (AMEKO, AE98327Ra; China). Following the manufacturer's instructions, the standard and sample solutions were transferred to a 96‐well plate coated with specific antibodies for NE or immunogenic derivatized H_2_S conjugate. After incubation at 37°C, the plate was washed, and horseradish peroxidase (HRP)‐labeled reagent was added. Following another incubation at 37°C and a subsequent wash, chromogenic solution was added sequentially, and the reaction was terminated to stop color development. The optical density (OD) was measured at 450 nm using an ELISA plate reader (MK3, Thermo Scientific, USA). NE levels were measured in triplicate for each sample, and the mean value was used for subsequent analysis. Fourteen biological replicates were performed. H_2_S levels were measured in triplicate for each sample, and the mean value was used for subsequent analysis. Four biological replicates were conducted. Based on the data, a linear regression curve was plotted with the concentration of the standard as the independent variable and the OD as the dependent variable. Using the equation and values from this curve as the y‐axis, the NE, or H_2_S concentration, in each sample was calculated.

### 2.7 Immunofluorescence analysis

The brain tissue after 4% polyformaldehyde fixation was rinsed with tap water, then dehydrated with ethanol using a gradient, made transparent with xylene, and then cut into 4‐μm‐thick coronal sections using an ultrathin microtome after wax impregnation and embedding. Sections were incubated with primary antibodies at 4°C overnight. The concentrations of primary antibodies were as follows: CBS (1:200; sc‐515180; Santa Cruz Biotechnology), p‐PERK (1:200; bs‐3330R; Bioss), TXNIP (1:100; ab232330; Abcam), NLRP3 (1:80; ab314905; Abcam), ASC (1:50; AF6234; Beyotime Biotechnology), and Caspase‐1 (1:100; sc‐56036; Santa Cruz Biotechnology). After three washes with PBS (5 min each), the sections were incubated with either Cy3‐conjugated Goat Anti‐Rabbit IgG (1:100, Boster, BA1032; China) or FITC‐conjugated Goat Anti‐Rabbit IgG (1:100, Boster, BA1105; China) for 1 h at 37°C in the dark. For the negative control, the primary antibody was replaced with antibody dilution buffer, while all other steps were performed identically to the experimental samples. Images were captured on a Leica fluorescence microscope (DFC350 FX camera; LAS V4.3) using a 10×/0.25 NA dry objective. Fluorescence signals were visualized with the following filter sets: CY3 (excitation 530–550 nm, emission 575–625 nm) and FITC (excitation 470–495 nm, emission 510–550 nm). Quantitative analysis of fluorescence intensity in the target brain region was performed using ImageJ.

### 2.8 Western blot analysis

After homogenizing each group of PVN tissues, the total protein was extracted from each group. Protein quantification was performed by detecting the protein concentration using a BCA protein concentration assay kit (Beyotime, P0012S; China). Subsequently, an appropriate amount of loading buffer was added, and the samples were stored in the refrigerator at −80°C. Protein samples and a pre‐stained marker were added sequentially to the comb wells in the vertical gel wells. Equivalent amounts of protein were separated by SDS‐PAGE and transferred to PVDF membranes (Boster, AR0136‐02; China). The membrane was sealed with BSA or skimmed milk powder for 1 h. Following the sealing, the membrane was exposed to the following primary antibodies at 4°C in the refrigerator overnight: anti‐β‐actin (1:1800; TA‐09; ZSGB‐BIO), CBS (1:1000; sc‐515180; Santa Cruz Biotechnology), PERK (1:2000; WL03378; WanLei), p‐PERK (1:2000; bs‐3330R; Bioss), TXNIP (1:1500; ab232330; Abcam), NLRP3 (1:2000; ab314905; Abcam), ASC (1:1000; AF6234; Beyotime Biotechnology), pro‐Caspase‐1 (1:1500; sc‐56036; Santa Cruz Biotechnology), Caspase‐1 p20 (1:2000; WL02996a; WanLei), IL‐1β (1:1000; ab234437; Abcam), TNF‐α (1:1000; ab215188; Abcam), and IL‐18 (1:2000; AF5207; Beyotime Biotechnology). Then, the membranes were incubated with the corresponding HRP‐conjugated anti‐rabbit (Boster, BA1054, China) or anti‐mouse IgG (Boster, BA1056, China) for 1 h at 37°C. The membranes were visualized and detected using enhanced chemiluminescence (ECL) solution (Boster, AR1170, China).

### 2.9 RT‐qPCR

Total RNA was extracted from cells of each group and PVN tissues of rats using RNAiso Plus reagent (Takara Bio, 9108; China). The concentration and purity of the RNA samples were assessed using an ultra‐micro spectrophotometer. The reaction solution was prepared following the instructions provided in the Takara kit (Takara Bio, 6110A; China), and the reaction conditions were adjusted to facilitate the reverse transcription of mRNA into cDNA. The reaction solution was prepared following the instructions provided in the Takara kit. It was then added to an octuple tube and immediately transferred to the real‐time fluorescence quantitative PCR instrument to protect it from light. The predetermined program for the RT‐qPCR reaction was set up. After confirming that the solubilization curve and amplification curve fell within the normal range, the expression of the target gene was calculated using the 2^−△△CT^ method, with β‐actin serving as the reference gene. For cell experiments, each assay was performed in three technical replicates, and the mean value was used for analysis, with three independent biological replicates. For animal experiments, each assay was performed in three technical replicates, and the mean value was used for analysis, with four independent biological replicates. The names and sequences of primers are shown in Table 1.

**TABLE 1:** Primer sequences used in this study.

Gene	Forward (5′–3′)	Reverse (5′–3′)
IL‐1β	TGCAGGCTTCGAGATGAAC	AGCTCATGGAGAATACCACTTG
TNF‐α	AGACCCTCACACTCAGATCA	GTCTTTGAGATCCATGCCATTG
IL‐18	CGACCGAACAGCCAACGAATCC	TCACAGATAGGGTCACAGCCAGTC
NLRP3	ACCTACGAAGCAATGCCCTT	GCAGTTGTCTAACTCCAGCAT
β‐Actin	AGTGGGATAACCTGGGCTCT	CAGGGTGGACTTGAACTGGT

### 2.10 Statistical analysis

SPSS 29.0 software was used for statistical analysis. Quantitative information followed a normal distribution and was presented as mean ± SD. One‐way ANOVA was used to compare multiple groups. LSD *t*‐test was used for multiple comparisons. Repeated measures information was analyzed using repeated measures ANOVA, and a significance level of *p* < 0.05 was considered statistically significant.

## 3 RESULTS

### 3.1 MAP measurement

**FIGURE 1:**
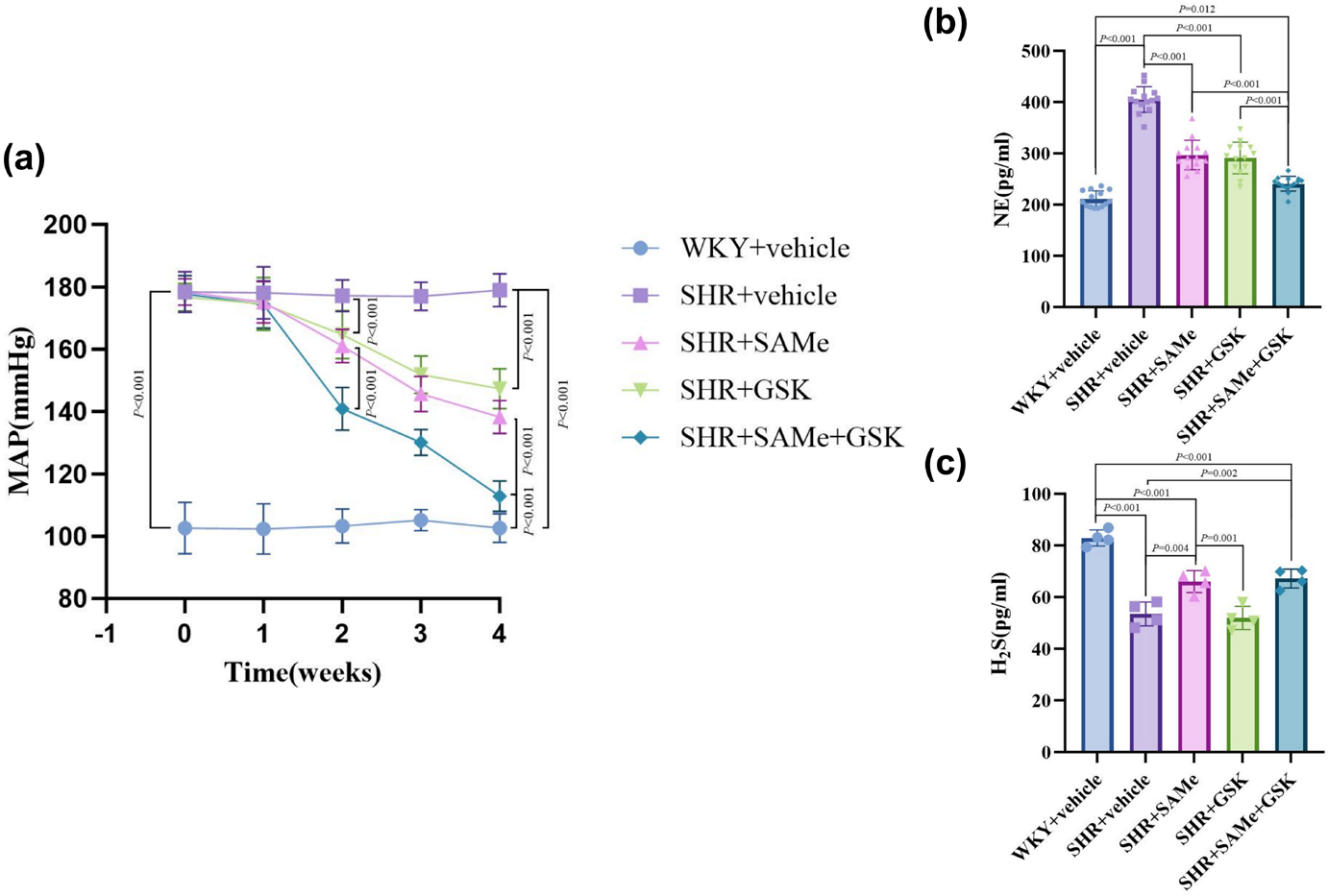
Effects of SAMe and GSK intervention on blood pressure, plasma NE levels, and PVN levels of H_2_S in SHR. (a) Changes in MAP. (b) Changes in plasma NE. (c) Changes in PVN levels of H_2_S. All data are expressed as a mean ± SD. *n* = 14 in each group for MAP and NE, *n* = 4 in each group for H_2_S. SAMe (an endogenous H_2_S agonist), GSK (a PERK inhibitor).

Before drug intervention, MAP was significantly higher in the SHR + vehicle group, SHR + SAMe group, SHR + GSK group, and SHR + SAMe+GSK group compared with the WKY + vehicle group (*p* < 0.01). MAP was consistently higher in the SHR + vehicle group than in the WKY + vehicle group during the intervention (*p* < 0.01). In the 2nd, 3rd, and 4th weeks of drug intervention, MAP was significantly decreased in both the SHR + SAMe group and the SHR + GSK group compared with the SHR + vehicle group (*p* < 0.01). Additionally, MAP was significantly decreased in the SHR + SAMe+GSK group compared with either the SHR + SAMe group or the SHR + GSK group (*p* < 0.01) (Figure 1a).

### 3.2 NE levels in plasma

Compared with the WKY + vehicle group, plasma NE levels in the SHR + vehicle group were increased (*p* < 0.01). plasma NE levels in the SHR + SAMe group and the SHR + GSK group were decreased compared to the SHR + vehicle group (*p* < 0.01). Furthermore, plasma NE levels in the SHR + SAMe+GSK group were decreased compared to the SHR + SAMe group or the SHR + GSK group (*p* < 0.01) (Figure 1b).

### 3.3 H_2_S levels and CBS expression in the PVN

**FIGURE 2:**
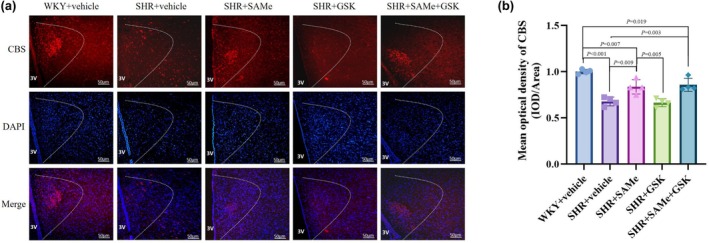
Effect of SAMe and GSK treatment on CBS expression in the PVN of SHR. (a) Immunofluorescence staining of CBS in the rat PVN, scale bar = 50 μm. (b) Statistical graph in average absorbance of CBS in the rat PVN. All data are expressed as a mean ± SD. *n* = 4 in each group. SAMe (an endogenous H_2_S agonist), GSK (a PERK inhibitor).

Compared with the WKY + vehicle group, the H_2_S level and CBS expression in the PVN of rats in the SHR + vehicle group decreased (*p* < 0.01); compared with the SHR + vehicle group, the H_2_S level and CBS expression in the SHR + SAMe group increased (*p* < 0.01), and the difference in the SHR + GSK group was not statistically significant (*p* > 0.05); compared with the SHR + SAMe group, the difference in H_2_S levels and CBS expression in the PVN of rats in the SHR + SAMe+GSK group was not statistically significant (*p* > 0.05) (Figures 1c, 2, 8a,b); No specific fluorescence signal was detected in the negative controls where the primary antibody was omitted (Figure S1), confirming the specificity of the immunostaining.

### 3.4 Expression of PERK/TXNIP/NLRP3 pathway‐related proteins in the PVN

**FIGURE 3:**
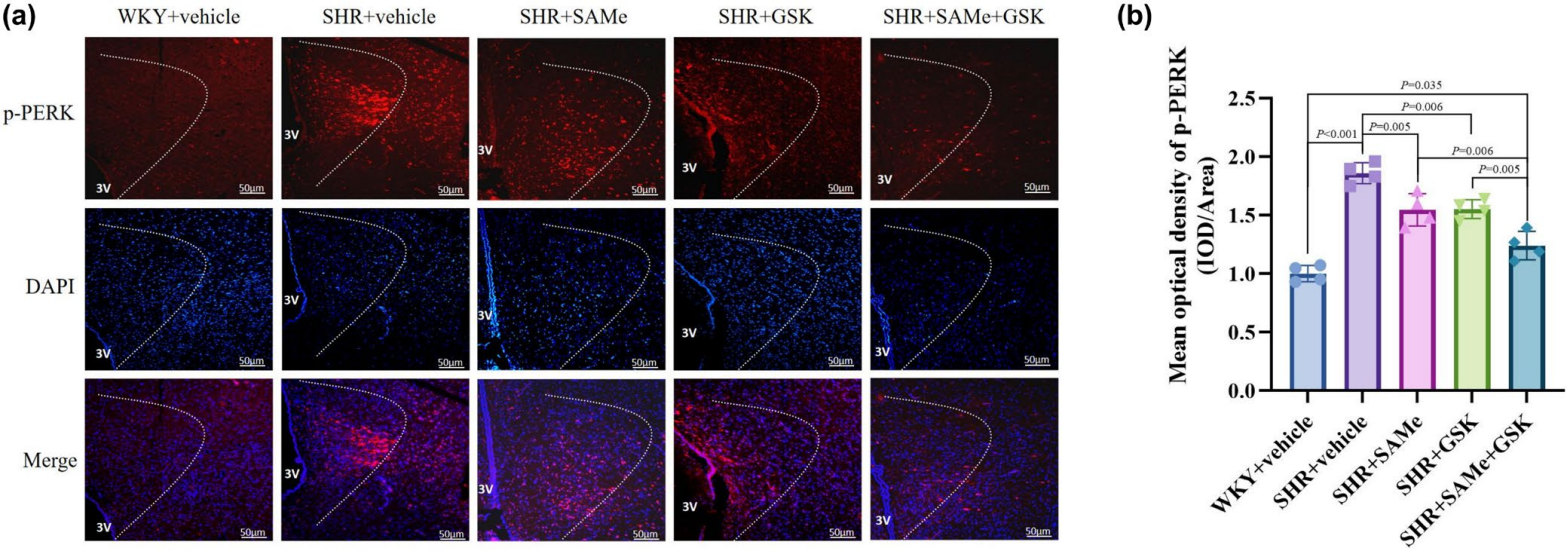
Effect of SAMe and GSK treatment on p‐PERK expression in the PVN of SHR. (a) Immunofluorescence staining of p‐PERK in the rat PVN, scale bar = 50 μm. (b) Statistical graph in average absorbance of p‐PERK in the rat PVN. All data are expressed as a mean ± SD. *n* = 4 in each group. SAMe (an endogenous H_2_S agonist), GSK (a PERK inhibitor).

**FIGURE 4:**
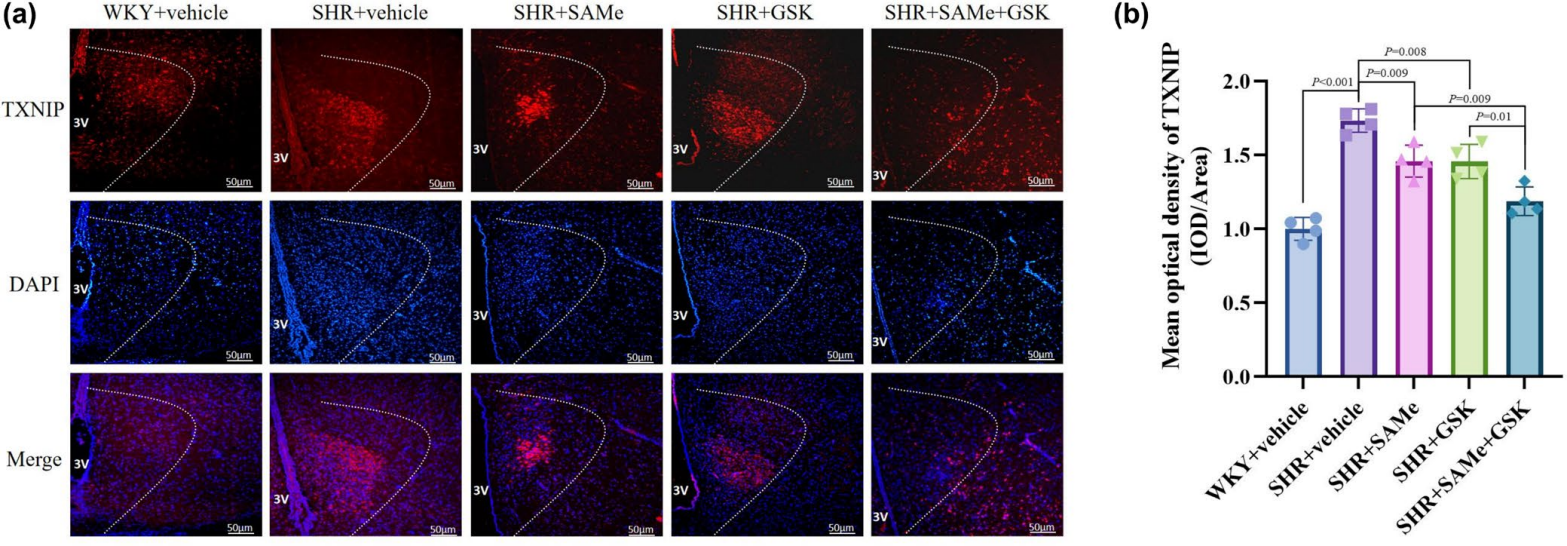
Effect of SAMe and GSK treatment on TXNIP expression in the PVN of SHR. (a) Immunofluorescence staining of TXNIP in the rat PVN, scale bar = 50 μm. (b) Statistical graph in average absorbance of TXNIP in the rat PVN. All data are expressed as a mean ± SD. *n* = 4 in each group. SAMe (an endogenous H_2_S agonist), GSK (a PERK inhibitor).

**FIGURE 5:**
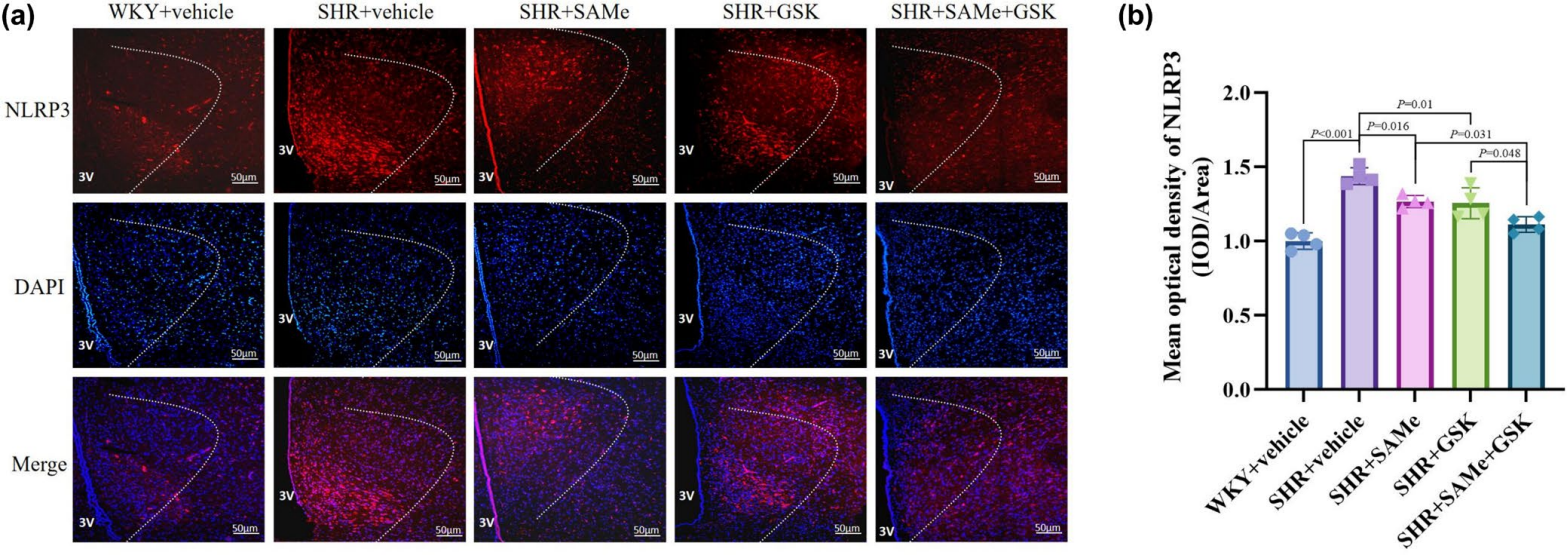
Effect of SAMe and GSK treatment on NLRP3 expression in the PVN of SHR. (a) Immunofluorescence staining of NLRP3 in the rat PVN, scale bar = 50 μm. (b) Statistical graph in average absorbance of NLRP3 in the rat PVN. All data are expressed as a mean ± SD. *n* = 4 in each group. SAMe (an endogenous H_2_S agonist), GSK (a PERK inhibitor).

**FIGURE 6:**
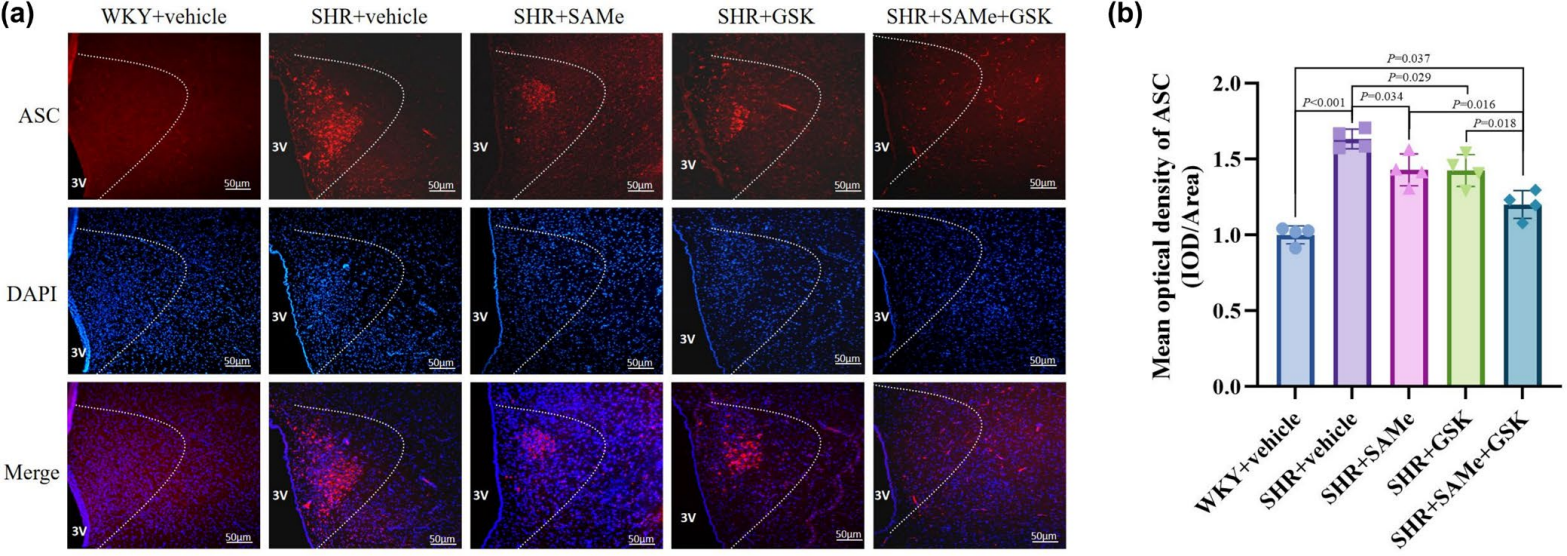
Effect of SAMe and GSK treatment on ASC expression in the PVN of SHR. (a) Immunofluorescence staining of ASC in the rat PVN, scale bar = 50 μm. (b) Statistical graph in average absorbance of ASC in the rat PVN. All data are expressed as a mean ± SD. *n* = 4 in each group. SAMe (an endogenous H_2_S agonist), GSK (a PERK inhibitor).

**FIGURE 7:**
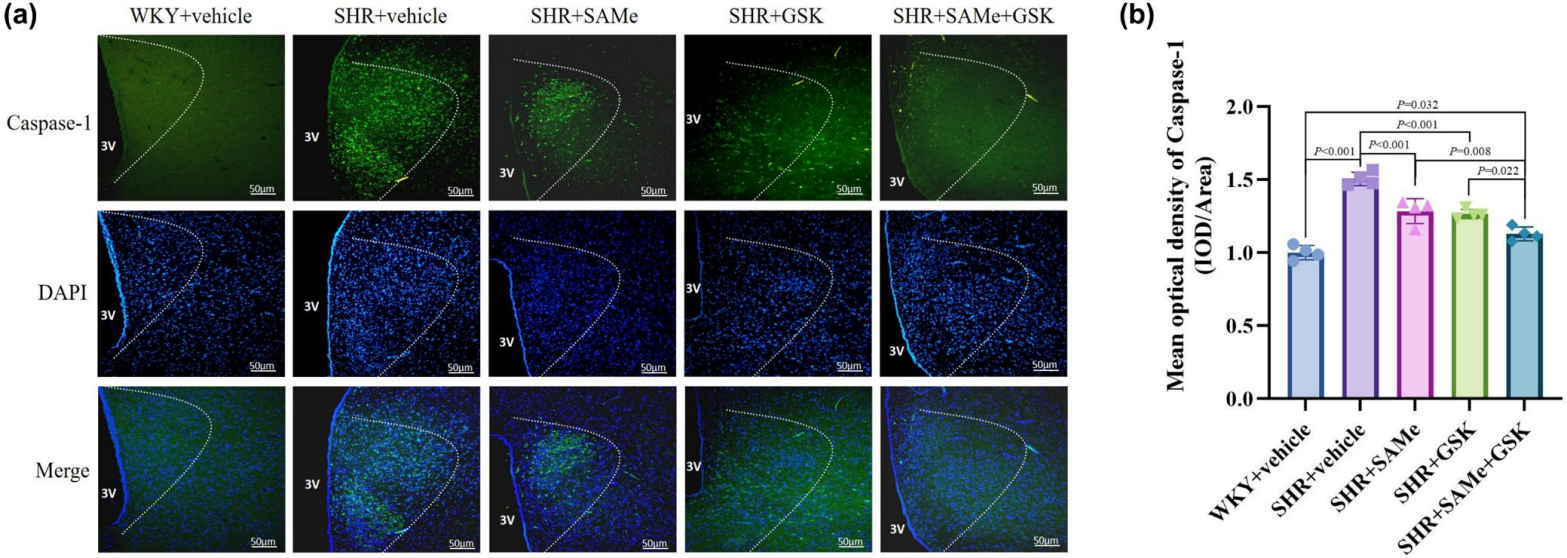
Effect of SAMe and GSK treatment on Caspase‐1 expression in the PVN of SHR. (a) Immunofluorescence staining of Caspase‐1 in the rat PVN, scale bar = 50 μm. (b) Statistical graph in average absorbance of Caspase‐1 in the rat PVN. All data are expressed as a mean ± SD. *n* = 4 in each group. SAMe (an endogenous H_2_S agonist), GSK (a PERK inhibitor).

**FIGURE 8:**
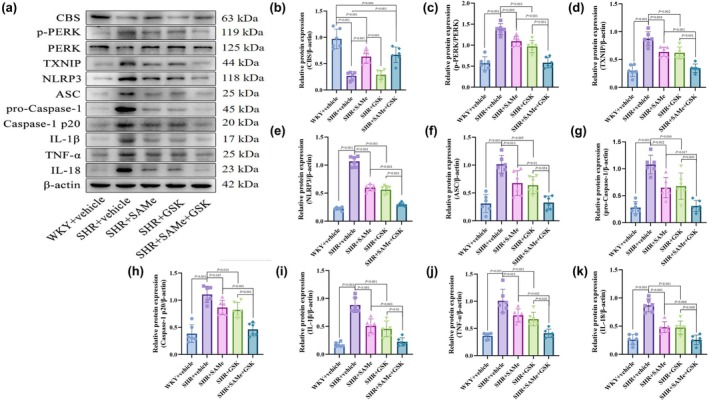
Effects of SAMe and GSK on the expression of CBS, PERK/TXNIP/NLRP3 pathway‐related proteins, and inflammatory cytokines in the PVN of SHR. (a) Protein electropherograms of CBS, p‐PERK/PERK, TXNIP, NLRP3, ASC, pro‐Caspase‐1, Caspase‐1 p20, IL‐1β, TNF‐α, and IL‐18. (b–k) Results of statistical analysis of the related proteins and inflammatory factors in the PVN of each group of rats, respectively. All data are expressed as a mean ± SD. *n* = 6 in each group. SAMe (an endogenous H_2_S agonist), GSK (a PERK inhibitor).

Compared with the WKY + vehicle group, the expression of p‐PERK, TXNIP, NLRP3, ASC, pro‐Caspase‐1, Caspase‐1 p20, IL‐1β, TNF‐α, and IL‐18 was significantly higher in the SHR + vehicle group (*p* < 0.01); compared with the SHR + vehicle group, the PERK/TXNIP/NLRP3 pathway in the SHR + SAMe group and the SHR + GSK group related protein expression was significantly lower (*p* < 0.05); compared with the SHR + SAMe group or SHR + GSK group, PERK/TXNIP/NLRP3 pathway‐related protein expression was significantly lower in the SHR + SAMe+GSK group (*p* < 0.05) (Figures 3, 4, 5, 6, 7 and 8a,c–k).

### 3.5 Expression of inflammatory factors in PVN

**FIGURE 9:**
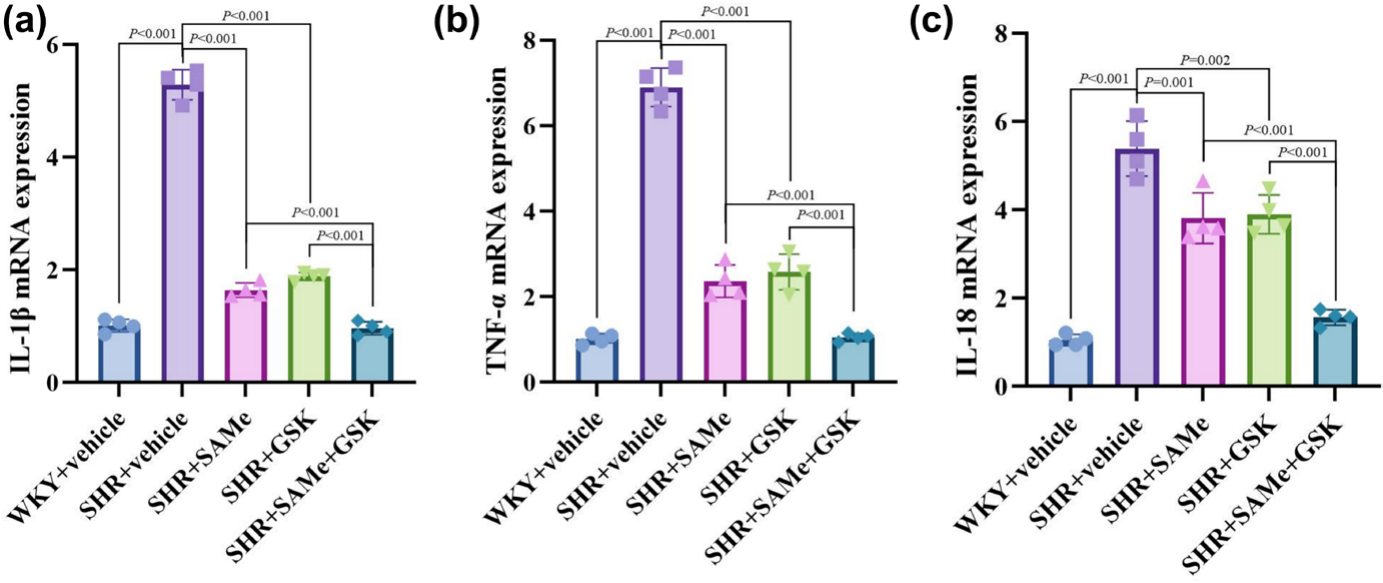
Effects of SAMe and GSK on the mRNA expression levels of IL‐1β, TNF‐α, and IL‐18 in the PVN of SHR. (a–c) Results of statistical analysis of mRNA levels of IL‐1β, TNF‐α, and IL‐18 in the PVN of rats in each group, respectively. All data are expressed as a mean ± SD. *n* = 4 in each group. SAMe (an endogenous H_2_S agonist), GSK (a PERK inhibitor).

The expression of IL‐1β, TNF‐α, and IL‐18 in the PVN of rats in the SHR + vehicle group was elevated compared with that in the WKY + vehicle group (*p* < 0.01); the expression of IL‐1β, TNF‐α, and IL‐18 in the PVN of rats in the SHR + SAMe and SHR + GSK groups was decreased compared with that in the SHR + vehicle group (*p* < 0.01); the expression of IL‐1β, TNF‐α, and IL‐18 in the PVN of rats in the SHR + SAMe + GSK group was decreased compared with the SHR + SAMe group or SHR + GSK group (*p* < 0.01) (Figure 9).

### 3.6 Cell viability assay

**FIGURE 10:**
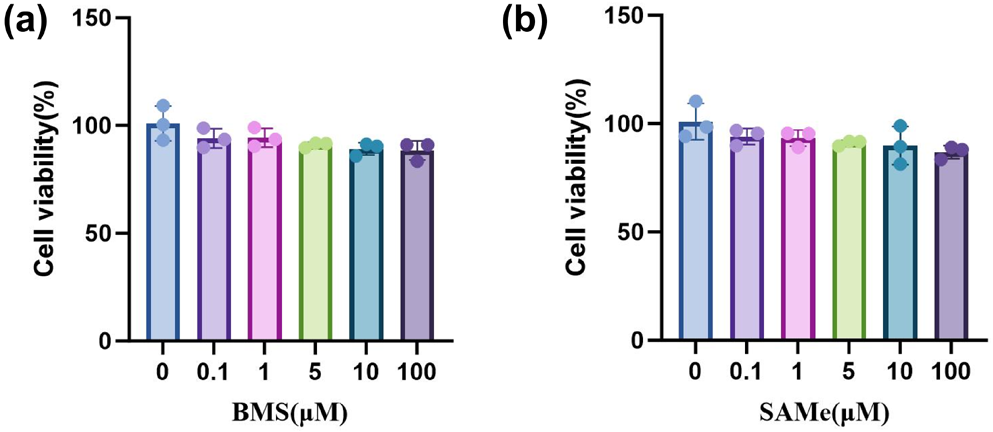
Effects of BMS and SAMe on cell viability of PC12 cells. (a) Results of the CCK‐8 assay in PC12 cells treated with BMS at concentrations of 0, 0.1, 1, 5, 10, and 100 μM. (b) Results of the CCK‐8 assay in PC12 cells treated with SAMe at concentrations of 0, 0.1, 1, 5, 10, and 100. All data are expressed as a mean ± SD. *n* = 3 in each group. BMS (an NLRP3 activator), SAMe (an endogenous H_2_S agonist).

In both SAMe and BMS groups, cell viability remained above 90% across the entire concentration range. Although a general decreasing trend was observed as concentrations increased, the change was not statistically significant (*p* > 0.05) (Figure 10). These results indicate that neither SAMe nor BMS exhibited significant cytotoxicity within the concentration range of 0.1–100 μM.

### 3.7 Expression of NLRP3 and inflammatory cytokines in cells

**FIGURE 11:**
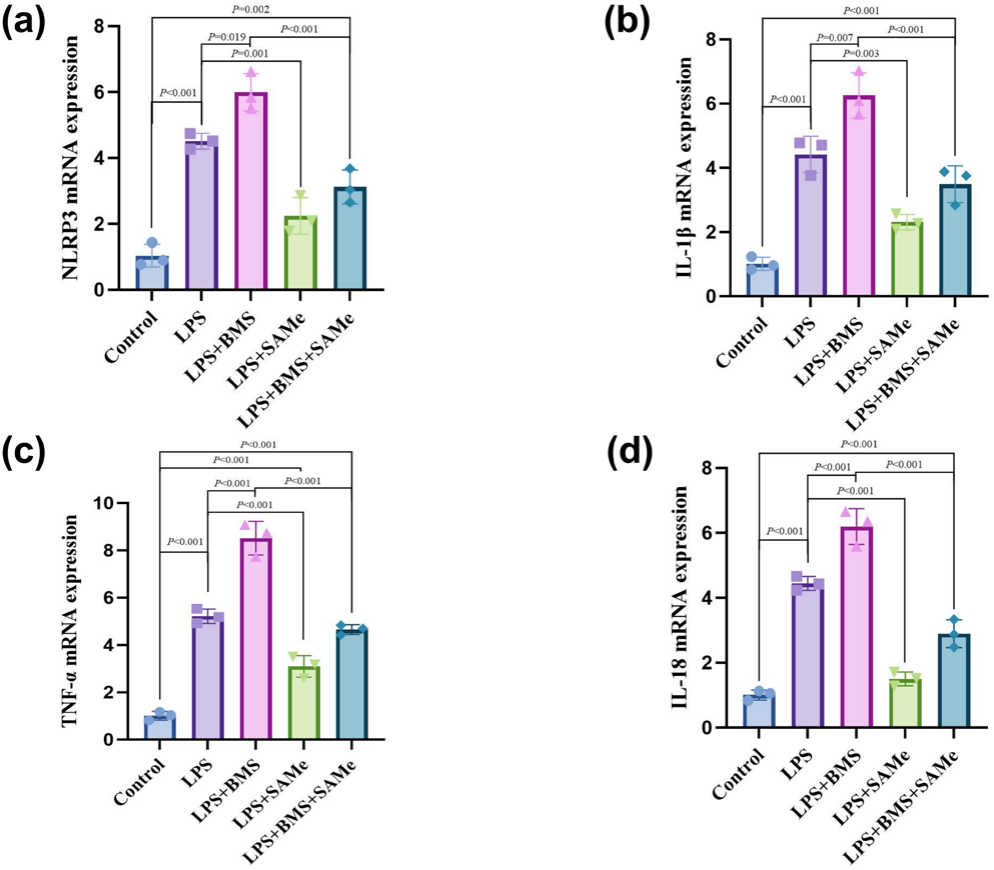
Effects of SAMe and BMS on the mRNA expression levels of NLRP3, IL‐1β, TNF‐α, and IL‐18 in PC12 cells. (a–d) Results of statistical analysis of mRNA levels of NLRP3, IL‐1β, TNF‐α, and IL‐18 in PC12 cells in each group, respectively. All data are expressed as a mean ± SD. *n* = 3 in each group. BMS (an NLRP3 activator), SAMe (an endogenous H_2_S agonist).

Compared with the control group, the expression of NLRP3, IL‐1β, TNF‐α, and IL‐18 was significantly elevated in the LPS group (*p* < 0.01). Compared with the LPS group, the expression of NLRP3, IL‐1β, TNF‐α, and IL‐18 was markedly reduced in the LPS + SAMe group (*p* < 0.01). In the LPS + BMS group, the expression of NLRP3, IL‐1β, TNF‐α, and IL‐18 was increased (*p* < 0.05). Additionally, compared with the LPS + BMS group, the expression of NLRP3, IL‐1β, TNF‐α, and IL‐18 was reduced in the LPS + BMS + SAMe group (*p* < 0.01) (Figure 11).

## 4 DISCUSSION

The present study demonstrated that appropriately increasing endogenous H_2_S levels in the PVN attenuates peripheral sympathetic nerve activity and reduces blood pressure in SHR. Mechanistically, this effect may be mediated through inhibition of the PERK/TXNIP/NLRP3 signaling pathway and subsequent suppression of neuroinflammation.

H_2_S is an endogenous gasotransmitter with diverse physiological functions, and its biosynthesis in the central nervous system is primarily catalyzed by CBS (Kolluru et al., 2023). Accumulating evidence has demonstrated that H_2_S plays a pivotal role in regulating blood pressure (Bełtowski & Kowalczyk‐Bołtuć, 2023; Wang, 2023). Hypertension and aging are known to affect the metabolism of sulfur‐containing compounds in the heart and kidneys, thereby reducing H_2_S production (Szlęzak et al., 2022). Microinjection of H_2_S donors or CBS activators into the RVLM of hypertensive rats has been shown to lower blood pressure by suppressing sympathetic outflow (Yu et al., 2015). Given that the RVLM receives axonal projections from the PVN (Akbari et al., 2024), a key cardiovascular regulatory center, we hypothesized that enhancing H_2_S production specifically within the PVN would ameliorate hypertension. Our study suggests that microinjection of SAMe, an endogenous CBS activator, into the PVN of SHR significantly increased local CBS expression and H_2_S levels, while also reducing plasma NE (a marker of sympathetic activity (Wang et al., 2025)) and MAP. These findings align with clinical observations that reduced NE levels are associated with decreased MAP in patients (Adda et al., 2021). Based on these results, we propose that reduced H_2_S levels in the PVN may contribute to the pathogenesis of hypertension, and that endogenous H_2_S may lower blood pressure by inhibiting peripheral sympathetic nerve activity.

PERK is one of the important proteins in the stress pathway of the endoplasmic reticulum (Ren et al., 2021). Studies have demonstrated that trimethylamine N‐oxide enhances Ang II‐induced vasoconstriction and hypertension through the PERK signaling pathway, while angiotensin‐(1‐9) inhibits Ang II‐induced hypertension via the same pathway (Guo et al., 2023; Jiang et al., 2021). Studies have shown that myocardial glutathione synthetase and TXNIP expression are significantly elevated in hypertensive and diabetic patients (Sklifasovskaya et al., 2023). The expression of NLRP3 inflammatory vesicles was notably elevated in the RVLM of rats with stress‐induced hypertension (Hu et al., 2020). The interaction between TXNIP and NLRP3 is considered one of the important regulatory mechanisms that enable the activation of NLRP3 inflammatory vesicles (Chen, Qiao, et al., 2022; Choi & Park, 2023). Notably, NLRP3 can be indirectly activated by PERK through the TXNIP protein (Jin et al., 2025). These findings suggest that activation of the PERK/TXNIP/NLRP3 signaling pathway may be involved in the pathogenesis of hypertension, and that targeting this pathway could exert antihypertensive effects. To test this hypothesis, we administered the PERK inhibitor GSK2606414 via bilateral injection into the PVN of SHR. The results showed that GSK2606414 inhibited PERK expression and significantly downregulated the expression of its downstream effectors. Concurrently, plasma NE levels and blood pressure were reduced. This indicates that the activation of the PERK/TXNIP/NLRP3 pathway might play a role in the onset of spontaneous hypertension. Inhibition of this pathway decreases peripheral sympathetic nerve activity and blood pressure.

H_2_S has regulatory effects on factors in the PERK/TXNIP/NLRP3 pathway in various diseases. Studies have demonstrated that H_2_S can reduce sepsis‐induced kidney injury through the PERK pathway (Song et al., 2024). Additionally, exogenous H_2_S alleviates ROS‐mediated endoplasmic reticulum stress, decreases the expression of endoplasmic reticulum stress marker proteins such as p‐PERK, mitigates apoptosis in septic cardiomyocytes, and effectively improves myocardial function (Zhao et al., 2022). By inhibiting the MAPK/TXNIP pathway, endogenous H_2_S shields against endothelial dysfunction (Tian et al., 2017). In addition, exogenous H_2_S inhibits the expression of NLRP3 inflammatory bodies by downregulating the ROS/TXNIP pathway, thereby improving non‐alcoholic fatty liver disease (Fu et al., 2025). H_2_S also prevents myocardium from hyperglycemia‐induced damage by inhibiting TXNIP‐mediated activation of NLRP3 inflammatory body complexes (Jia et al., 2020). However, whether H_2_S lowers blood pressure through this pathway in the setting of spontaneous hypertension remains unclear. To further elucidate this effect, we administered SAMe into the PVN of SHR and found that it significantly reduced the expression of PERK/TXNIP/NLRP3 pathway‐related proteins in the PVN, concomitant with decreases in MAP and peripheral sympathetic nerve activity. Notably, co‐administration of SAMe and GSK2606414 produced a significantly greater therapeutic benefit in SHR, characterized by greater decreases in blood pressure and downregulation of PVN pathway‐related proteins. This suggests that the paraventricular nucleus H_2_S has a regulatory effect on the PERK/TXNIP/NLRP3 pathway. Increasing endogenous H_2_S levels in the paraventricular nucleus reduces peripheral sympathetic nerve activity and lowers blood pressure, possibly by down‐regulating the PERK/TXNIP/NLRP3 pathway expression.

The inflammatory response of neuronal nuclei in the central nervous system is one of the important pathogenetic mechanisms of hypertensive disorders. Studies have shown that increased secretion of pro‐inflammatory cytokines in the central nervous system can directly activate sympathetic nerves (Yu et al., 2022). The blood pressure, sympathetic nerve activity, and heart rate were significantly increased after microinjection of TNF‐α and IL‐1β into the SFO (Wei et al., 2015). In a rat model of renal hypertension, there was an increased production of pro‐inflammatory cytokines TNF‐α, IL‐1β, and IL‐18 in the brain. Puerarin could lower blood pressure and provide neuroprotection by inhibiting the TLR4/NF‐κB‐mediated inflammatory response (Gao et al., 2024). The above findings show that central pro‐inflammatory cytokines might promote the development of hypertension. In recent years, a large number of studies have confirmed that H_2_S plays important pathophysiological roles in the anti‐inflammatory, neurological, and cardiovascular systems (Huang et al., 2023; Rodkin et al., 2023). Exogenous H_2_S inhibits NLRP3 and activates autophagy, reducing the levels of pro‐inflammatory cytokines such as IL‐1β and TNF‐α (Liu et al., 2021). H_2_S can inhibit NLRP3 inflammasome‐mediated neuroinflammation in diabetic encephalopathy (Ma et al., 2025). Previous studies by our group have found that exogenous H_2_S inhibits the expression of the inflammatory cytokine IL‐1β protein in the PVN and promotes the production of the anti‐inflammatory cytokine IL‐10. This regulation aids in the control of peripheral sympathetic nerve activity, which ultimately results in a reduction of blood pressure (Liang et al., 2024). This study found that the increase in endogenous H_2_S has a significant effect on the expression of inflammatory factors in the PVN of SHR rats and could improve the inflammatory response. When SAMe was combined with GSK2606414, the improvement in inflammation was markedly superior to that achieved with SAMe alone. Cellular experiments demonstrated that LPS induction significantly upregulated the expression of NLRP3 and pro‐inflammatory cytokines in PC12 cells, while SAMe intervention effectively reversed these abnormal expressions. More importantly, BMS‐986299 activation of NLRP3 eliminated the anti‐inflammatory effects of SAMe, further confirming that H_2_S exerts its anti‐inflammatory effects by targeting NLRP3 inflammasomes. This further validates the consistency of H_2_S‐mediated anti‐inflammatory mechanisms in both in vivo and in vitro models. The above analysis indicates that activation of the PERK/TXNIP/NLRP3 pathway in the PVN can induce inflammatory responses. Additionally, the inhibitory effect of H_2_S on the PERK/TXNIP/NLRP3 pathway in the PVN contributes to alleviating inflammatory responses and reducing blood pressure.

We concluded that decreased levels of H_2_S in the PVN and activation of the PERK/TXNIP/NLRP3 pathway may be crucial mechanisms in the pathogenesis of spontaneous hypertension. Increasing endogenous levels of H_2_S in the PVN inhibits peripheral sympathetic nerve activity and improves blood pressure in SHR rats. The regulatory mechanism may be associated with reducing the expression of the PERK/TXNIP/NLRP3 pathway in the PVN. This study only provided correlative evidence for the involvement of the PERK/TXNIP/NLRP3 pathway using pharmacological interventions with SAMe and GSK, without establishing causality via gene knockdown or knockout approaches. Additionally, given that PERK is a key upstream factor in the endoplasmic reticulum stress pathway, whether the interaction between inflammation and endoplasmic reticulum stress contributes to the development and progression of hypertension was not examined in this study. Therefore, future studies employing gene knockdown or knockout techniques are required to further elucidate the causal role of the PERK/TXNIP/NLRP3 pathway in hypertension and to investigate the specific molecular mechanisms underlying the interaction between inflammation and endoplasmic reticulum stress in the pathogenesis and progression of this disease.

## AUTHOR CONTRIBUTIONS

Dong‐Dong Zhang and Yan‐Feng Liang conceptualized and supervised the study. Shu‐Yue Chen, Hui Wang, Han‐Jun Song, Bao‐Cheng Zhang, Ming‐Fu Wang, Chao Lv, and Xiao‐Yu Wang performed all experiments. Shu‐Yue Chen, Hui Wang, Shuai Zhang, Huai‐Yu Xing, and Quan Yuan analyzed research data and performed statistical analysis. Shu‐Yue Chen, Hui Wang, Hong‐Bin Li, Hong‐Bin Li, Zhang‐Ran Xing, and Xin‐Yi Han drafted the manuscript. Dong‐Dong Zhang, Yan‐Feng Liang, Shu‐Yue Chen, Hui Wang, Han‐Jun Song, Bao‐Cheng Zhang, Ming‐Fu Wang, Chao Lv, Xiao‐Yu Wang, Shuai Zhang, Huai‐Yu Xing, Quan Yuan, Hong‐Bin Li, Xin‐Rui Ding, Zhang‐Ran Xing, and Xin‐Yi Han edited the manuscript. All authors reviewed the final manuscript.

## FUNDING INFORMATION

This research was funded by Natural Science Foundation of Heilongjiang Province (NO. PL2024H001), Excellent innovation team based on the basic scientific research vocational cost for the provincial undergraduate universities in Heilongjiang (NO. 2021‐KYYWF‐0595), North Medicine and Functional Food Characteristic Subject Project in Heilongjiang Province (NO. HLJTSXK‐2022‐03), Heilongjiang Province college students innovation and entrepreneurship training project (NO. S202310222008), Jiamusi University National Fund cultivation project (NO. JMSUGPZR2023‐015), Research Team of Immune Microenvironment and Major Chronic Diseases (2024‐KYYWF‐0617), “East Pole” Academic Team Project of Jiamusi University (DJXSTD202404).

## CONFLICT OF INTEREST STATEMENT

The authors declare no conflicts of interest.

## ETHICS STATEMENT

The animal study protocol was approved by the Jiamusi University Laboratory Animal Ethics Committee (protocol code JDJCYXY20240060 and date of approval 16 September 2021).

## Supporting information


Figure S1.

## Data Availability

The datasets generated during and analyzed during the current study are available from the corresponding author on reasonable request.
