# Supplementary material for: Mechanism of paraventricular nucleus H2S on PERK/TXNIP/NLRP3 pathway in male spontaneously hypertensive rats
Source: Physiol Rep. 2026 Apr 10;14(7):e70861. doi: 10.14814/phy2.70861 (PMC13069160; doi:10.14814/phy2.70861)

Negative control

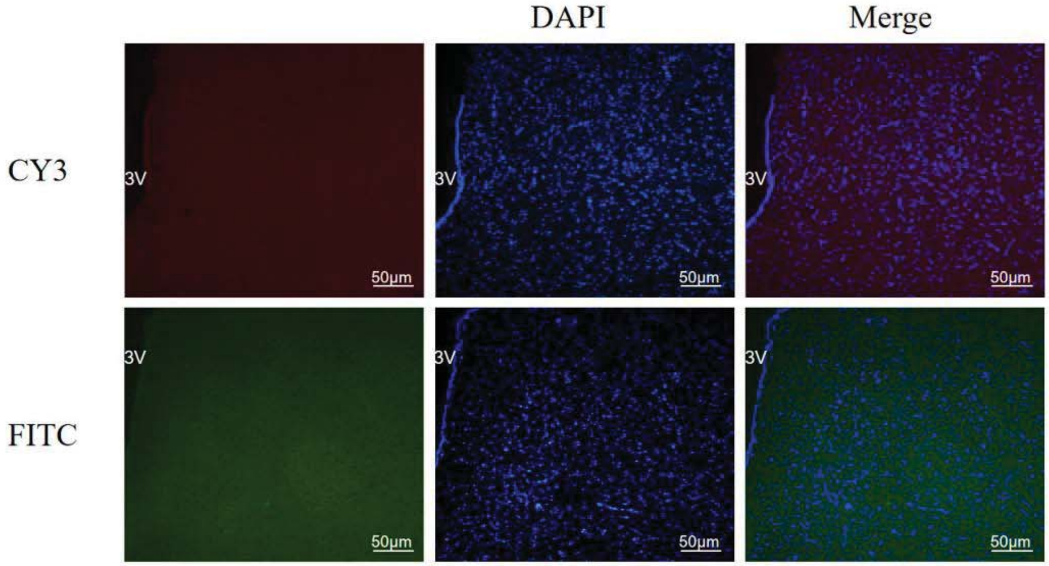

Fig. S1. Negative controls for immunofluorescence staining.
(A) CY3-conjugated Goat Anti-Rabbit IgG, scale bar = 50 μm.
(B) FITC-conjugated Goat Anti-Rabbit IgG, scale bar = 50 μm.

Original western blot for CBS

CBS 63kDa

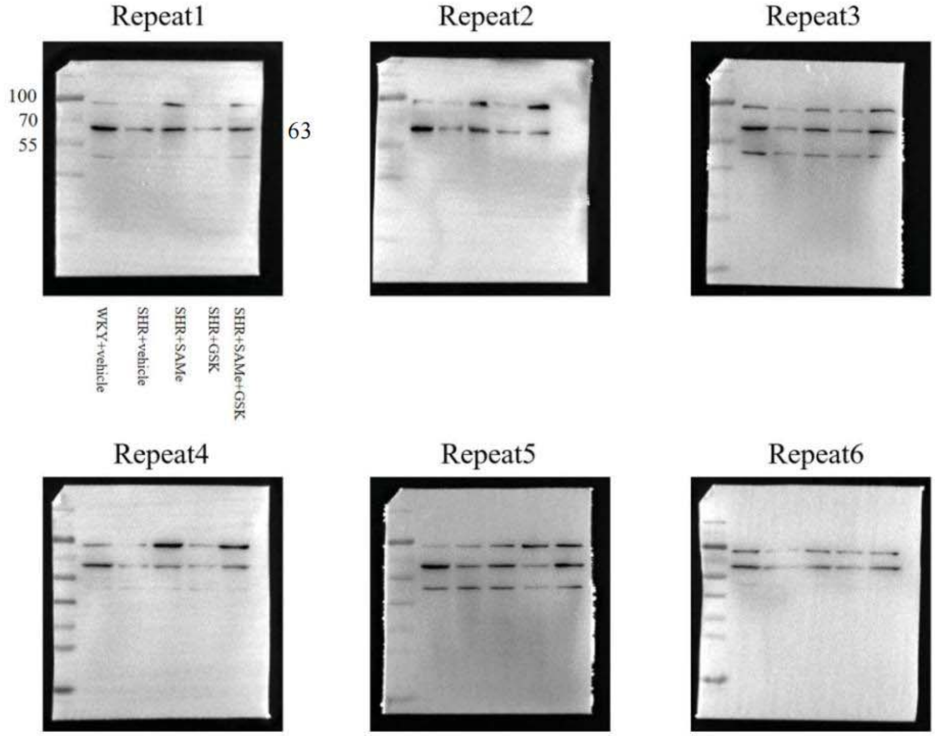

Original western blot for p-PERK

p-PERK 119kDa

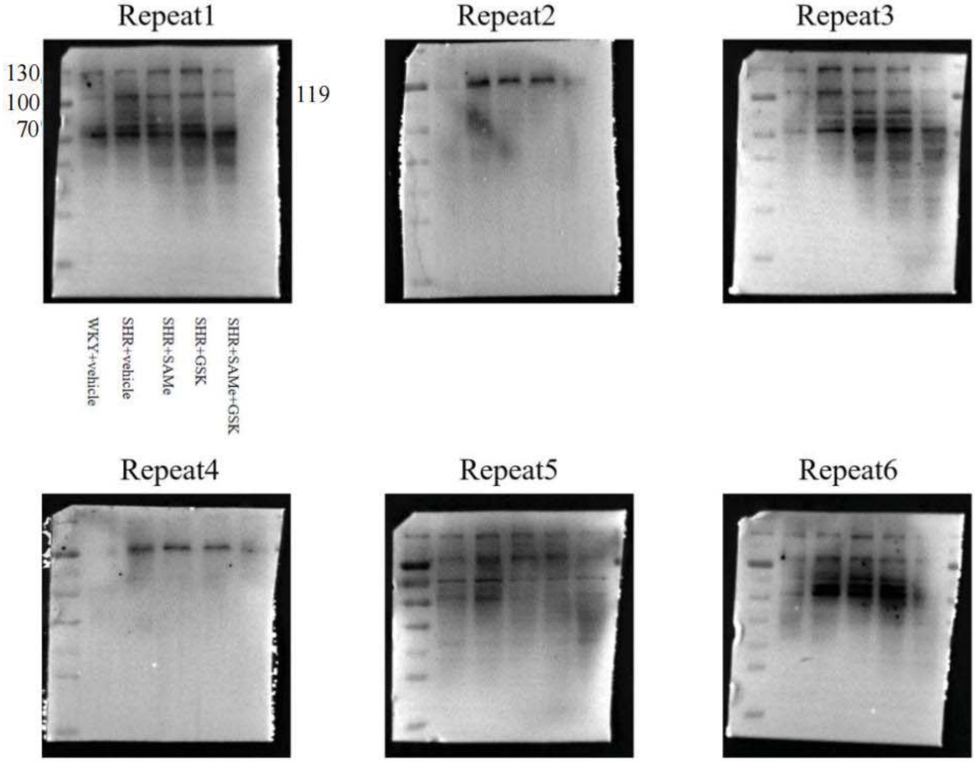

Original western blot for PERK

PERK 125kDa

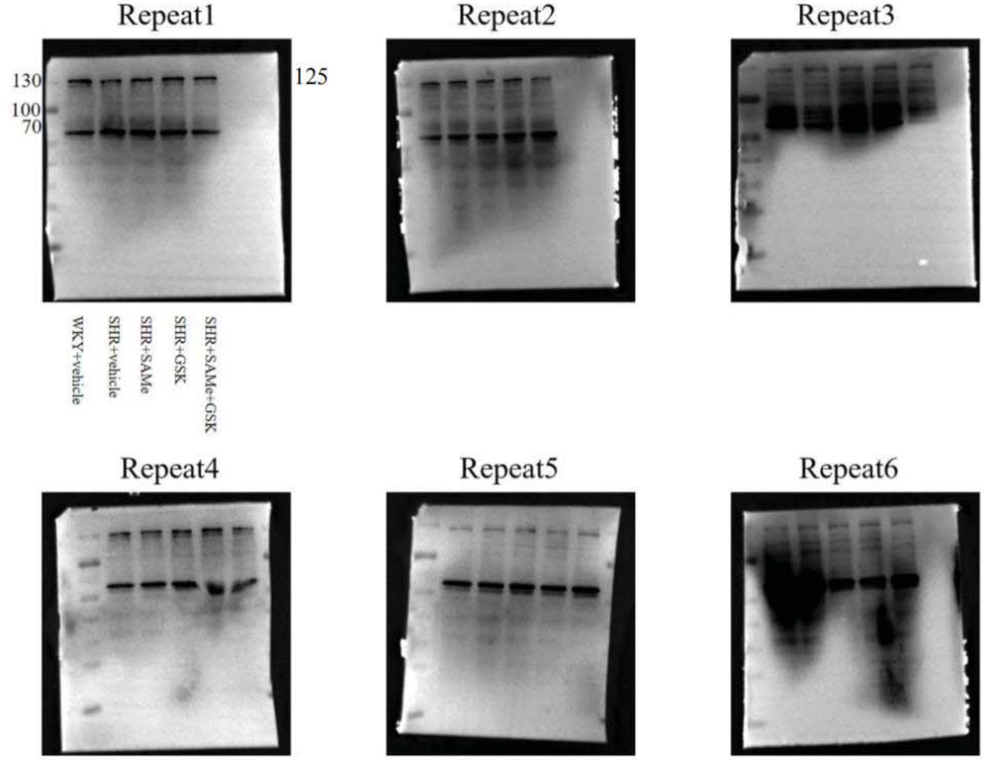

Original western blot for TXNIP

TXNIP 44kDa

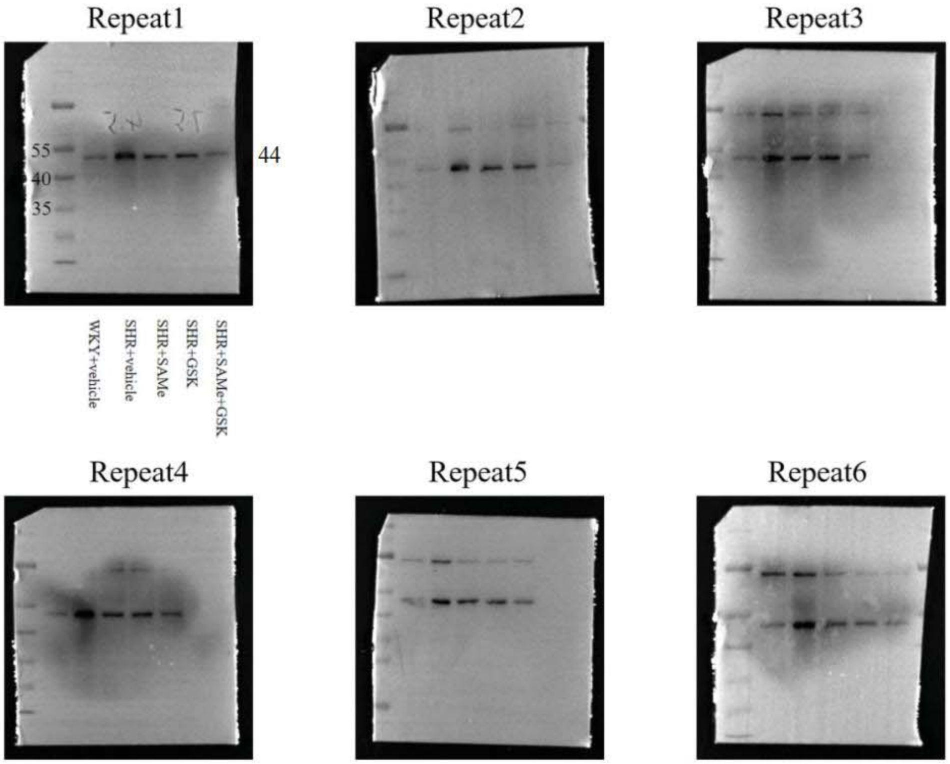

Original western blot for NLRP3

NLRP3 118kDa

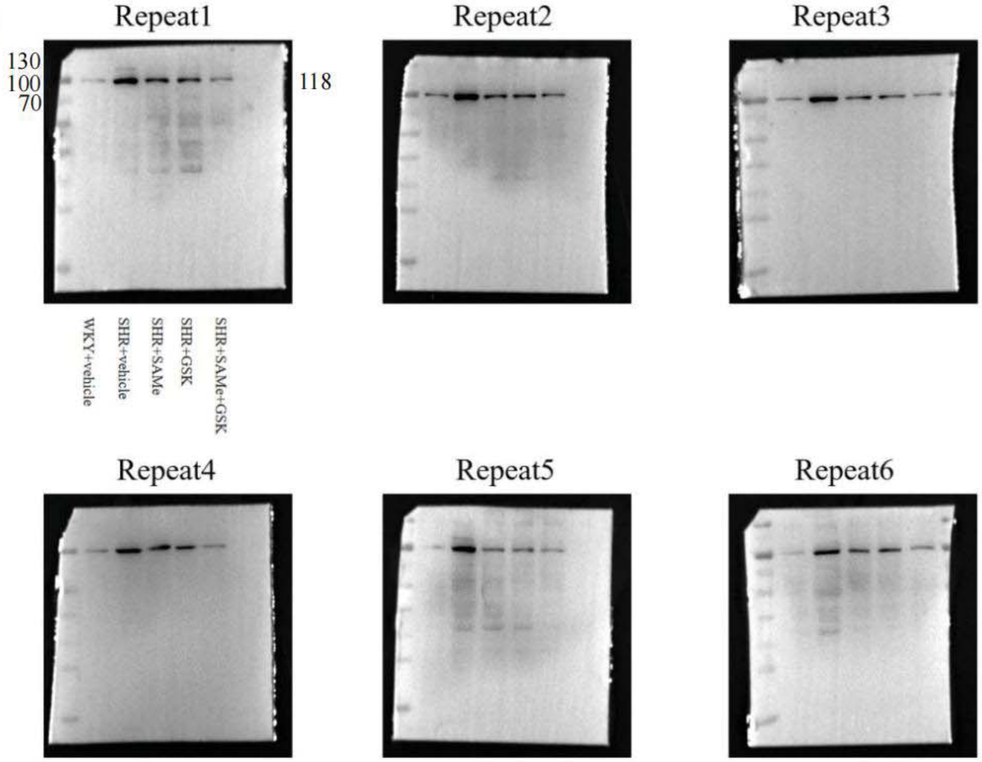

Original western blot for ASC

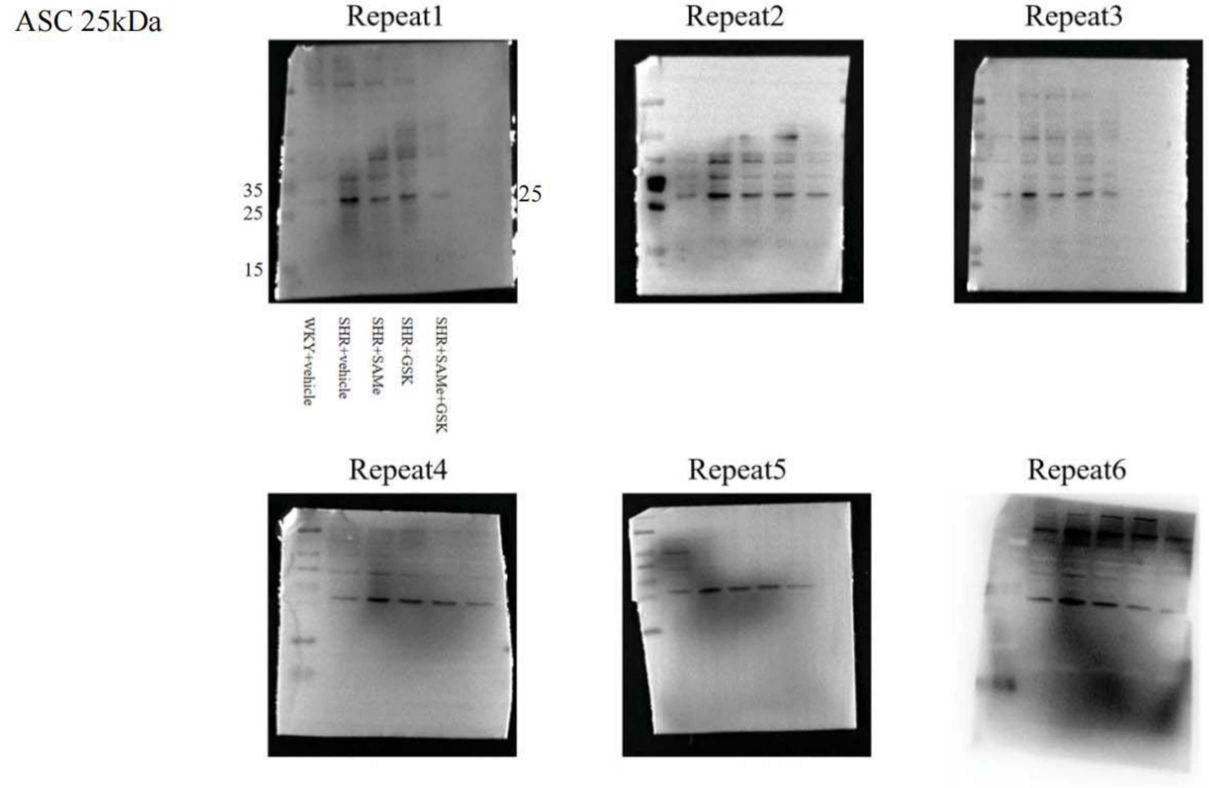

Original western blot for pro-Caspase-1

pro-Caspase-1 45kDa

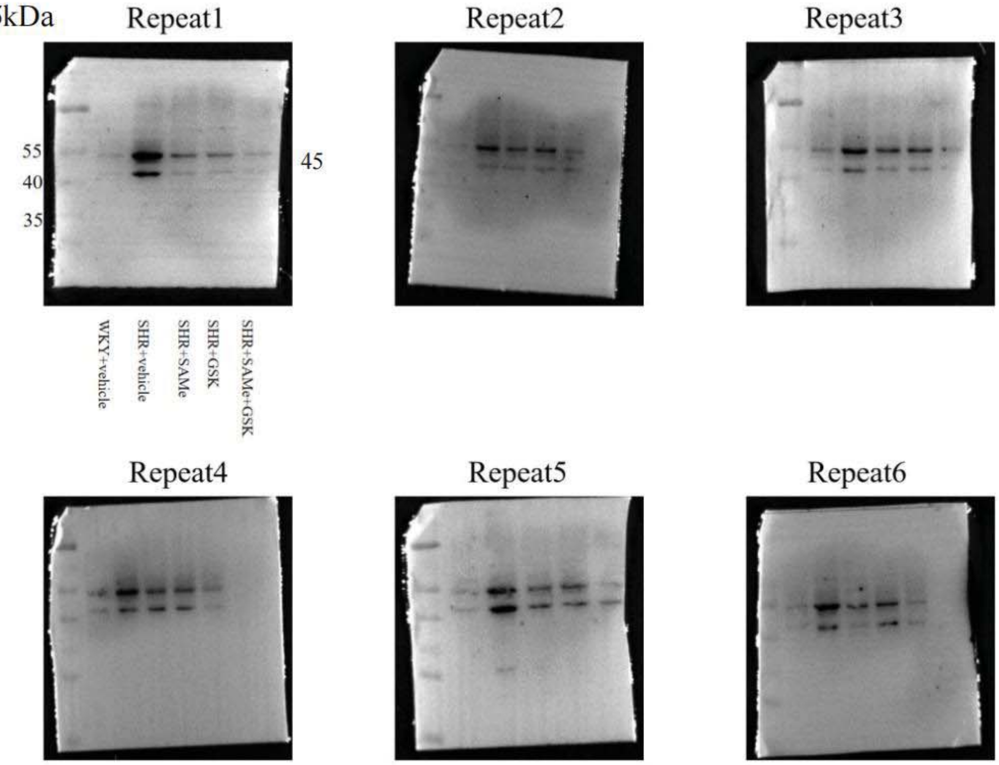

Original western blot for Caspase-1 p20

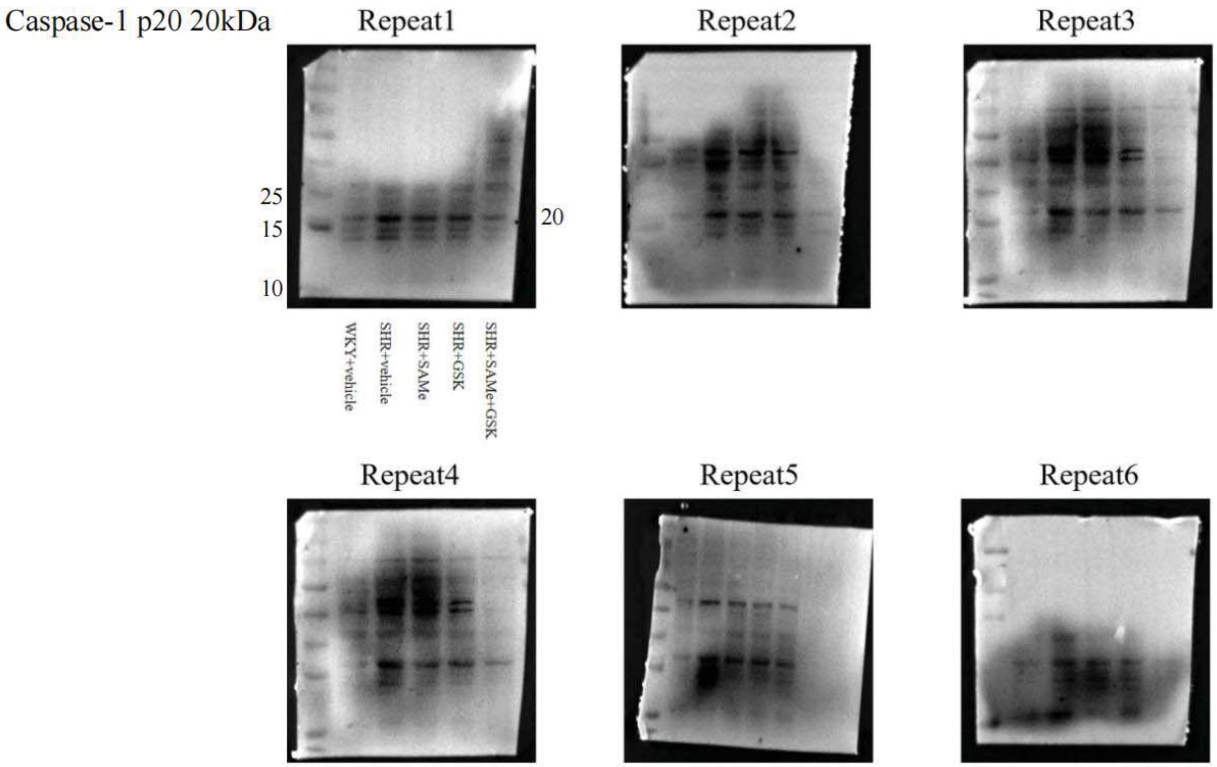

Original western blot for IL-1β

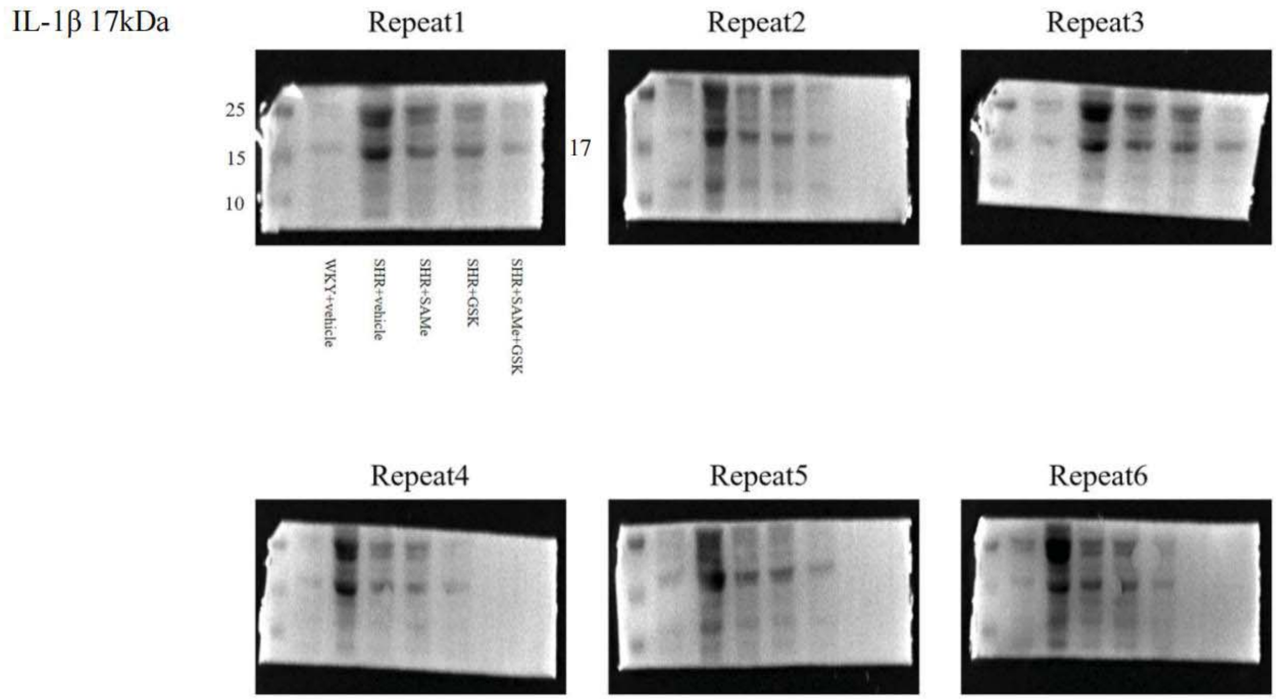

Original western blot for TNF-α

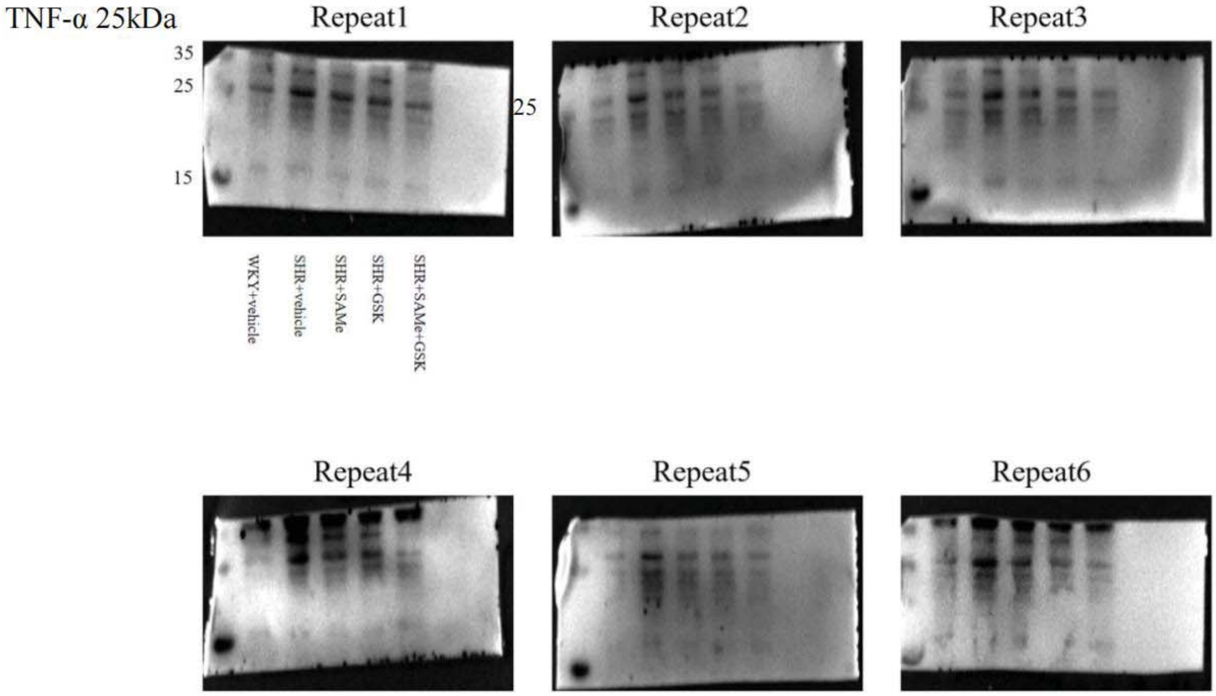

Original western blot for IL-18

IL-18 23kDa

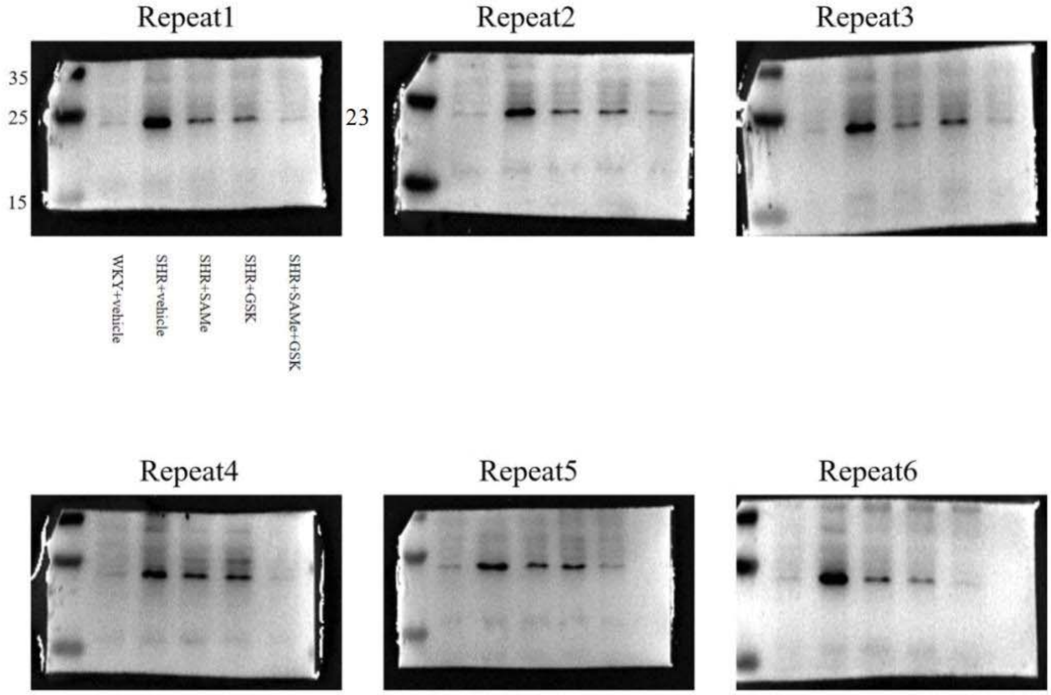

Original western blot for β-actin

β-actin 42kDa

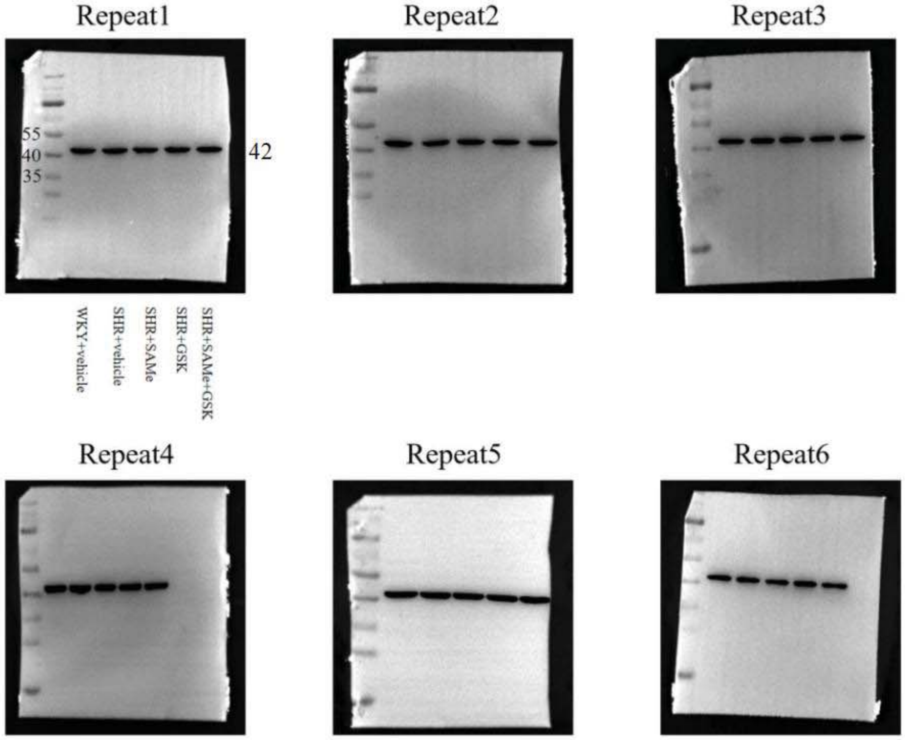

Supplement: Supplementary file 1 — Figure S1. [file PHY2-14-e70861-s001.pdf]
